# Biogenic gold nanoparticles synthesized from *Pergularia daemia* leaves: a novel approach for nasopharyngeal carcinoma therapy

**DOI:** 10.1515/biol-2025-1239

**Published:** 2025-12-30

**Authors:** Xijun Zhang

**Affiliations:** Department of Otolaryngology-Head and Neck Surgery, The Fourth Affiliated Hospital of Harbin Medical University, No. 37 Yiyuan Street, Nangang District, Harbin, 150001, China

**Keywords:** *Pergularia daemia*, AuNPs, apoptosis, proliferation, nasopharyngeal carcinoma

## Abstract

Gold nanoparticles (AuNPs) were biogenically synthesized using the aqueous leaf extract of *Pergularia daemia*, with process optimization achieved via response surface methodology. The resulting nanoparticles were monodispersed, crystalline, and exhibited an average diameter of 110 ± 2.8 nm. Fourier-transform infrared (FTIR) spectroscopy confirmed the presence of flavonoids, terpenoids, and polyphenols as capping and stabilizing agents, contributing to the nanoparticles’ colloidal stability for over six months. *In vitro* studies demonstrated significant dose and time-dependent antiproliferative effects of the biosynthesized AuNPs against human nasopharyngeal carcinoma (C666-1) cells, with the IC_50_ decreasing from 26.8 μg/mL at 24 h to 16.44 μg/mL at 48 h. Microscopic examination revealed marked cytoplasmic damage, and mitochondrial dysfunction was confirmed via DAPI and AO/EtBr staining. RT-PCR analysis revealed a substantial upregulation of pro-apoptotic genes Bax, Caspase-3, and p53, alongside downregulation of the anti-apoptotic protein Bcl-2, confirming apoptosis induction via the intrinsic mitochondrial pathway. Importantly, the Pd-AuNPs exhibited selective cytotoxicity toward carcinoma cells over normal NP69 cells, highlighting their potential as a targeted therapeutic agent for nasopharyngeal carcinoma.

## 1 Introduction

Cancer globally ranks as the second most common cause of mortality in nations with developed economies. The Southeast Asian region and South China have an elevated incidence of nasopharyngeal carcinoma (NPC), a cancer that develops from the nasopharyngeal epithelia [1], 2]. Known for its aggressive invasiveness and hidden position, almost 70 % of those suffering from NPC are first diagnosed with locoregionally advanced NPC, which has a dismal 5-year survival rate [3], 4]. Although standard concurrent chemoradiotherapy has produced a significant increase in survival, 20–30 % of patients at higher risk did not respond to treatment due to distant metastases and/or local–regional recurrence [5], 6]. Consequently, improved treatment approaches are desperately needed to increase the effectiveness of therapy for patients diagnosed with advanced NPC.

Recently, innovative and revolutionary therapies have been developed by using nanomaterials to regulate biological processes. There are now innumerable research projects in the discipline of cancer-focused medicine due to the development of nanotechnology. The emergence of nanotechnology offers the chance to create sub-micron nanocarriers, including liposomes, nanoparticles, and cubosomes, which are ideal for targeted drug administration and fall within the spectrum of 1–100 nm [7]. Nanoparticles may interfere with the targeted cells’ ability to function because of their increased penetrative capabilities due to their sub-micron dimensions [8]. There is a longer duration of accumulation and subsequent incorporation of these nanoparticles into bodily tissues [9]. Considerable emphasis has been paid to the development of novel and economical nanomaterials for the restoration of the environment, pollution monitoring, and additional applications. According to recent research, nanoparticles, nanofiltration, and other products arising from the development of nanotechnology might significantly address or improve many of the issues related to water quality [10], 11]. Over the past few decades, gold nanoparticles (AuNPs) have garnered a lot of attention because of their unique optical, electrical, and molecular discrimination abilities. According to Albert et al. [12], these nanoparticles have demonstrated significant promise in a number of biological functions, including drug delivery, imaging, sensing, diagnostics, and photothermal treatment.

AuNPs are less cytotoxic and more biocompatible compared to various metallic nanoparticles because of their inert characteristics [13]. AuNPs are traditionally synthesized chemically by reduction reactions [14] or physically via evaporation-condensation and laser treatment [15]. Using these techniques is frequently linked to several difficulties, such as high expense, high energy and time consumption, labour intensity, and toxicity concerns. As a result, new methods for producing AuNPs safely need to be developed. Recently, environmentally friendly, low-cost, nontoxic, and highly active nanoparticles have been created using green synthesis methods.

Actually, a variety of biomaterials, including plant parts, bacteria, fungi, and algae, are employed as reducing agents in the environmentally friendly production of nanoparticles [16]. Furthermore, because plant extracts are widely available, need little upkeep, are biosafe, and are reasonably priced, they provide several advantages over alternative biological methods [17]. Due to the combined impacts of plant metabolites and nanogold, sustainable nanoparticles are not only more affordable and ecologically friendly than chemically manufactured ones, but they also frequently show higher biological activity [18]. [Several herbal extracts were used to quickly and effectively synthesise AuNPs extracellularly [19].

The common perennial climbing herb *Pergularia daemia* (forsk, family Asclepiadaceae) has great therapeutic significance and is often employed in conventional therapies to treat a variety of human ailments. Among their many pharmacological properties, conventional therapy has shown hepatoprotective, cancer-preventing, anti-diabetic, anti-inflammatory, antioxidant, and cardiovascular benefits [20], [21], [22], [23], [24]. Modern studies have found a wide variety of medicinally useful compounds, especially glycosides. However, the literature has not yet explored the potential of *P. daemia* for producing green AuNPs. Plants can produce AuNPs, though Pd-AuNPs produced with *P. daemia* offer unique advantages. They are specifically cytotoxic to nasopharyngeal carcinoma (NPC) cells while leaving healthy epithelial cells intact. They may also induce apoptosis via the intrinsic mitochondrial pathway through their phytochemicals with bioactive properties. Our investigation, which is the first to examine the development of environmentally friendly AuNPs agent NPC cells with molecular evaluation, demonstrates the potential of *P. daemia* as a novel therapeutic strategy.

Numerous studies have demonstrated the encouraging anticancer properties of plant-mediated AuNPs towards a range of tumor cell lines, including ovarian, liver, cervical, and breast cancers [25], 26]. In recent years, a variety of biogenic metal nanoparticles, including copper, magnesium, zinc, and gold-based formulations, have demonstrated promising anticancer potential against multiple tumour models. For example, Cu_4_O_3_ nanoparticles have shown efficacy against ovarian and cervical cancer cells [27], 28], while MgO nanoparticles exhibited activity against breast cancer [29], 30]. Biogenic Cu–Mn bimetallic nanoparticles derived from plant extracts have also been explored for their cytotoxic effects [31], 32], and ZnO nanoparticles have been reported to induce apoptosis in breast cancer cells [33]. Additionally, gold and silver nanoparticles synthesized through green methods have demonstrated selective cytotoxicity across various cancer cell lines [34], 35]. These studies collectively highlight the versatility and therapeutic potential of biogenic metal nano-formulations, supporting their further development as anticancer agents and providing context for the present investigation on Pd-AuNPs. It was determined that the anticancer properties were relatively harmless to normal cells, dose-related, and specific [36], 37]. Due to their improved penetration and retention impact, AuNPs preferentially accumulate in tumor tissues because of their permeable vasculature [38], 39]. The most prevalent head and neck cancer, nasopharyngeal carcinoma (NPC), has been the subject of just a few studies assessing the cytotoxic ability of green AuNPs [40]. Several etiologic factors contribute to the development of NPC, and the fundamental cellular processes are yet unknown. Therefore, new, safer, and more effective treatment drugs are desperately needed to combat NPC.

The goal of this work is to use *P. daemia* leaf extract to create a green synthesis procedure for AuNPs. Both the anticancer properties and biocompatibility of the nanoparticles can be improved by the adsorbed phytonutrients. The study also intends to comprehensively assess the therapeutic properties of biogenic-mediated *P. daemia* AuNPs (Pd-AuNPs) on the human NPC cell line *in vitro*. In spite of the biological potential of AuNPs documented in a number of publications, our study is unique in that it targets a rare malignant nasopharyngeal cancer with few available treatments and uses *P. daemia*, a conventionally significant medicinal plant, for nanoparticle synthesis. Crucially, we show preferential cytotoxicity against cancer cells in contrast to normal NP69 cells and offer molecular insights into apoptosis induction via the intrinsic mitochondrial pathway. The suggested green nanosystem may therefore be a viable option for targeted nasopharyngeal carcinoma treatment.

## 2 Materials and methods

### 2.1 Preparation of *P. daemia* extract

To shield the thermo-labile element in *P. daemia* from the damaging effects of direct sunlight, fresh leaves had been rinsed with pristine water and allowed to dry at ambient temperatures. The dried-up leaves were physically crushed using a mixer-blender. After that, 5 g of *P. daemia* leaves powder were weighed and added to a 250 mL conical flask with 180 mL of distilled water. They were then stirred with a magnetic stirrer and cooked for 5 min using a heating mantle. After passing through a muslin cloth and filter paper (Whatman No. 1), the aqueous extract was kept at 4 °C for additional usage. A refrigeration unit was used to store the filtrate for the biogenesis of gold nanoparticles.

### 2.2 Biogenic production of gold nanoparticles

To produce AuNPs biogenically, *P. daemia* and gold (III) chloride trihydrate (HAuCl_4_·3H_2_O) were combined in a 2:1 ratio in 20 mL of double-distilled water at 70–80 °C. The resulting mixture was sonicated for half an hour till it turned dark purple instead of yellow. Following that, the mixture was centrifuged for 15 min at 15,000 rpm. The dried powder was kept at 4 °C for later usage after the extracted AuNPs had been dissolved in double-distilled water.

### 2.3 Characterization of biogenic AuNPs

#### 2.3.1 Absorption of the UV-visible spectrum

Using a UV–Visible spectroscopy in the 300–700 nm wavelength range, the production and stability of biogenic AuNPs were examined. The production of nanoparticles and the color shift were noted. At ambient temperatures (RT), fresh samples were used for the spectroscopic investigation.

#### 2.3.2 FT-IR spectroscopy

A Fourier-transform infrared spectrophotometer (FTIR) was used to investigate biogenic AuNPs in the 400–4,000 cm^−1^ frequency spectrum. This device provides the highest resolution of 0.5 cm^−1^, a neighbourhood of 2,100 cm^−1^, a 30,000:1 ratio, and a 1-min aggregate. Before FTIR analysis, AuNPs were purified by spinning and reconstituting them in sterilized water.

#### 2.3.3 FE-SEM and EDX analysis

A Field Emission Scanning Electron Microscope (FE-SEM) functioning at a voltage range of 20–25 kV was used to rapidly compute the morphological characteristics of the AuNPs produced from the *P. daemia* extract. The material’s composition was analyzed using EDX (energy-dispersive X-ray) spectroscopy.

#### 2.3.4 X-ray diffraction spectrum (XRD)

The XRD technique was recently used to ascertain the structure of AuNPs. Dried biogenic gold nanoparticles were applied to the XRD grid (XPERT-PRO) for X-ray diffraction (XRD) investigations, and scattering was observed during 2 h between 20 and 80 °C. At 40 kV and 40 mA of electrical current, the spectrum information was acquired.

#### 2.3.5 DLS and zeta potential analysis

Biogenic AuNPs’ dimensions and dispersion characteristics were assessed using the Shimadzu IG-1000 plus dynamic light scattering (DLS) particle size analyzer. After mixing the sample with water and sonicating it for 20 min, it underwent evaluation.

### 2.4 Cell culture and maintenance

Normal nasopharyngeal epithelial cells (NP69) and a human nasopharyngeal cancer cell line (C666-1 cells) were obtained from the Institute of Biochemistry and Cell Biology, Chinese Academy of Sciences (Shanghai, China). The cell bank routinely authenticates these lines using short tandem repeat (STR) profiling and ensures they are free of mycoplasma contamination. Cells were cultivated in RPMI 1640 at 37 °C in a humidified environment with 95 % air and 5 % CO_2_. Cells from passages 5–15 were used in all studies. Cells were seeded onto 96-well plates at an average density of 5 × 10^4^ cells/well for cytotoxicity assessments, and they were left to adhere for 24 h before treatment.

### 2.5 MTT assay

A previously presented procedure was used to investigate the cytotoxicity of biogenic Pd-AuNPs towards C666-1 and NP69 cells [41]. Different concentrations of biogenically synthesized Pd-AuNPs were administered to cancer cells, and the cells were then incubated for 24 and 48 h at 37 °C. Incubation time was limited to 24 and 48 h since longer periods (72 h) could cause non-specific cell death and nutrition depletion, which could confuse the precise impact of Pd-AuNPs. After incubation, cells were washed with phosphate-buffered saline, and the culture medium was discarded. The plate was then incubated at 37 °C for 4 h in a dark place after 15 μL of MTT reagent (0.5 mg/mL) was added to each well. 150 μL of DMSO was transferred to every well after 4 h, and its absorbance at 570 nm was thereafter determined. This equation was used to determine the proportion of viable cells:

Percentage of viability=absorbance of treated cells/ absorbance of control cells×100.

The IC_50_ values were calculated by nonlinear regression analysis using a four-parameter logistic model (GraphPad Prism 9.1). The regression equation applied was:

$$Y=\text{Bottom}+\left(\text{Top}-\text{Bottom}\right)/{1+10}^{\left(\log\;{\text{IC}}_{50}-X\right)\;}\times \text{Hillslope}$$

This model provided the IC_50_ values of 26.8 μg/mL (24 h) and 16.44 μg/mL (48 h) for C666-1 cells.

### 2.6 Clonogenic survival assay

A sufficient number of duplicate C666-1 cells were planted and cultured in six-well plates. The cells underwent treatment with biogenic Pd-AuNPs at IC_50_ values and vehicle control (0.1 % DMSO) when they reached 70 % confluence. They were then incubated for seven days at 37 °C (minimum 50 cells/colony). Following the removal of the medium, the cells were given a single PBS wash, followed by the addition of 2–3 mL of fixation solution and a 5-min incubation period at the ambient temperature. Following fixation, the cells were cultured for 2 h at ambient temperature, and then stained using a 0.5 % crystal violet mixture. After that, the crystal violet was thrown away, the cells were cleaned with tap water, and the stereomicroscope was used to count the colonies that were formed [42]. The overall number of colonies in the absence of Pd-AuNPs was set as 100 % to compute the proportion of colony establishment. The mean ± SD (*n* = 3) is used to express the values. *p < 0.05 was regarded as substantial data, in contrast to pretreated control cells.

### 2.7 Intracellular ROS generation

To investigate how the biogenic produced Pd-AuNPs led to the production of ROS in C666-1 cells, DCFH-DA staining was employed. DMEM was used to load cells at an average density of 1 × 10^6^ on a 24-well plate, and the cells were kept at 37 °C for 24 and 48 h. Following a 24 and 48-h incubation period, the cells were exposed to IC_50_ concentrations of Pd-AuNPs. After adding 10 μL of DCFH-DA dye to each well, the wells were left for an additional hour. Following many PBS washes, the internal ROS content was measured using a flow cytometer.

### 2.8 DAPI staining

By using the subsequent DAPI labelling approach on cancer cell lines, the apoptosis was also thoroughly examined. The dye produces luminous colours with ds-DNA. Here, the apoptotic cells’ nuclei exhibit both deep staining and fragmentation with shorter chromatin.

### 2.9 Mitochondrial membrane potential (MMP)

The effect of biogenic fabrication of Pd-AuNPs on the MMP importance in C666-1 cells was examined using Rh-123 labeling [43]. At a density of 1 × 10^5^ cells, the C666-1 cells were cultured in DMEM at 37 °C for 24 and 48 h. The C666-1 cell culture was subsequently enhanced with Pd-AuNPs at an IC_50_ dosage. After that, the mixture was maintained for at least one more day at 37 °C. After adding 10 μg/mL of Rh-123 to each well, the HeLa cells were stained for half an hour. Ultimately, a fluorescent microscope was used to examine the MMP site of pretreated C666-1 cells.

### 2.10 AO/EB staining

The efficacy of Pd-AuNPs to induce apoptosis in C666-1 cells was examined using AO/EB simultaneous imaging. 4 × 10^5^ cells were introduced into a 24-well plate and exposed to air for 24 and 48 h at 37 °C. Following the administration of IC_50_ dosages of Pd-AuNPs to the C666-1 cells, the cells were colored by adding 100 μg/mL of AO/EB dye in a 1:1 ratio to each well and allowed to settle for 5 min. Finally, using a fluorescent microscope to see treated cells, apoptosis was discovered.

### 2.11 Apoptosis analysis

The proportion of cells that underwent apoptosis was determined by flow cytometric evaluation using the Annexin V detection kit (Immunostep, Spain) in accordance with the directions provided by the company. Following being collected and rinsed with PBS, C666-1 cells were briefly resuspended in 1× Annexin-binding buffer. Following that, this suspension was combined with the Annexin-V/PI staining solution. The samples were examined after being incubated in dark conditions for 15 min. According to PI, the gating technique for annexin-V interpretation is applied to dot plots of forward side scatter (FSC) and side scatter (SSC), which show how stain fluorescence is expressed on cells.

### 2.12 Cell cycle analysis

C666-1 cells were seeded at a count of 1 × 104 cells/plate for the cell cycle study, which was earlier reported [43]. Following that, the cells underwent exposure to Pd-AuNPs at IC_50_ concentrations for 24 and 48 h. After centrifugation, trypsinization, and a cold PBS wash, the cells were gathered in vials. The pellet was then combined with 70 % ethanol and ice-cold PBS, and the end result was maintained at −20 °C for approximately 1 h. The tubes were then centrifuged, and the cell pellet and 1× PI (propidium iodide (PI) (Sigma, USA), PBS, and RNase A) were added. The entire mixer configuration has been incubated for 15 min at ambient temperature. The cell cycle distribution was then investigated using flow cytometry in compliance with the manufacturer’s guidelines (FACS, BD Biosciences). Three tests were conducted on each sample.

### 2.13 Quantitative real-time PCR

The mRNA expression of apoptosis and anti-apoptotic genes was examined in C666-1 cancer cells exposed to Pd-AuNPs. A commercially accessible RNAEasy kit (Qiagen, Hilden, Germany) was used to separate the RNA from the control and treated cells. The isolation procedures were followed according to the manufacturer’s guidelines. A Nanodrop spectrophotometer was used to test and evaluate the RNA’s purity and level. The apoptotic and anti-apoptotic genes Bax, Bcl-2, caspase-3, and p53 were analyzed using the SYBR Green PCR kit. Data on the relative intensity of the targeted genes was gathered and examined. The PUBMED sequence about earlier research provides the primer designs for the desired genes (Table 1). The 2^−ΔΔCT^ formula was used to express the comparable transcripts of the target biomarkers’ mRNA expressions, which were normalized to the baseline gene GAPDH.

**Table 1:** Primer sequences used for quantitative real-time PCR.

Gene	Forward primer (5′→3′)	Reverse primer (5′→3′)
Bax	TTT​GCT​TCA​GGG​TTT​CAT​CC	CAG​TTG​AAG​TTG​CCG​TCA​GA
Bcl-2	GGT​GGG​GTC​ATG​TGT​GTG​G	CGG​TTC​AGG​TAC​TCA​GTC​ATC​C
Caspase-3	AGA​ACT​GGA​CTG​TGG​CAT​TGA​G	GCT​TGT​CGG​CAT​ACT​GTT​TCA​G
p53	CCA​GCC​AAA​GAA​GAA​ACC​ACT​G	TCT​GTA​CGG​CGG​TCT​CTC​CAG
GAPDH	GAA​GGT​GAA​GGT​CGG​AGT​C	GAA​GAT​GGT​GAT​GGG​ATT​TC

### 2.14 Western blotting analysis

To determine the protein expression, C666-1 cells were plated at a density of 5 × 10^4^ cells/plate. The cells were exposed to 26.8 μg/mL Pd-AuNPs for 24 and 48 h at 37 °C. After the cells were harvested, RIPA buffer containing the following components was used to lyse the cells: 50 mM Tris–HCl, pH 8.0, 0.5 % sodium deoxycholate, 1 mM EDTA, 150 mM NaCl, 0.1 % SDS, and 1 % NP-40. The protein content was then determined using the PierceTM BCA protein assay kit (Thermo Scientific). Following the preparation of SDS-PAGE, a 10 μg quantity of protein was added. It was filtered, and the gel was transferred to PVDF membranes. The transfected membranes underwent blocking with 5 % bovine serum albumin (BSA; Thermo Fisher Scientific) for 2 h at ambient temperature before being exposed for an entire night at 4 °C with diluted (1:1,000) primary antibodies. The membranes were rinsed with 1× TBST solution before being probed for 2 h at ambient humidity using the proper horseradish peroxidase-conjugated secondary antibody (1:5,000). The blots were further processed employing an ECL (electro-chemiluminescence) detection system (Bio-Rad Laboratory, USA) following a 1× TBST wash. The protein densitometry was evaluated using the ImageJ tool.

### 2.15 Analytical statistics

GraphPad Prism 9.1 and Excel were used to analyze the data. Plotting of the drug dosage needed to 50 % inhibit the growth of cells (IC_50_) was done employing the PRISM program and non-linear regression evaluation with dose-response graphs. All experiments were performed in triplicate, with at least three independent biological replicates. Data are presented as mean ± standard deviation (SD). Statistical significance was determined using one-way ANOVA followed by Tukey’s post-hoc test, with *p* < 0.05 considered significant. Error bars shown in the figures indicate SD from three independent experiments.

## 3 Results and discussion

### 3.1 Biogenic fabrication of Pd-AuNPs using *P. daemia* extract

**Figure 1:**
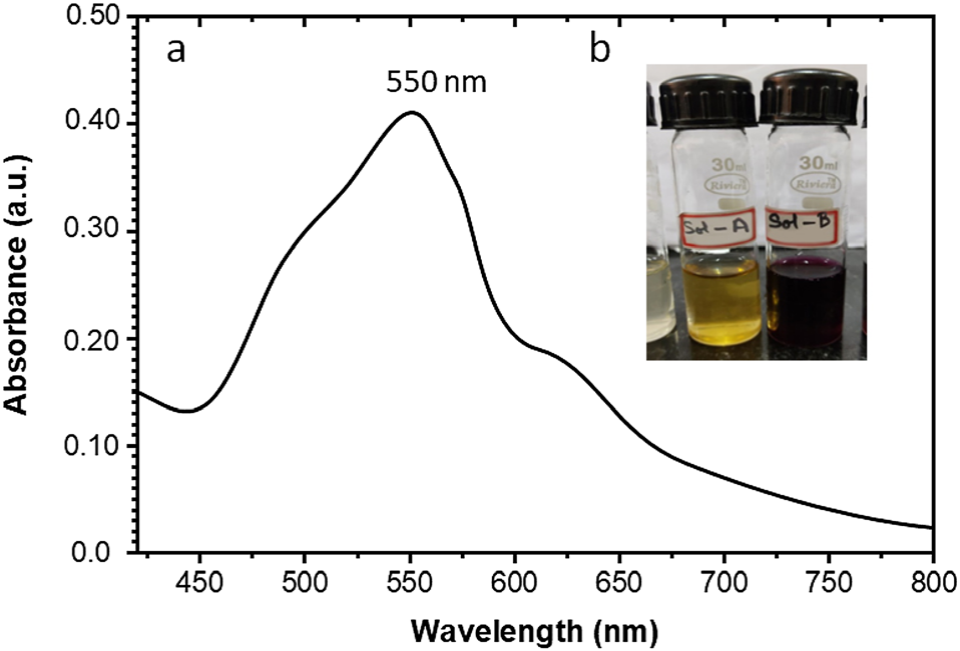
Characterization of Pd-AuNPs. (a) UV–vis spectra of biogenic gold nanoparticles developed from an extract from *Pergularia daemia*. (b) In the presence of *P. daemia* extract, Pd–AuNP fabrication is shown by a colour shift from yellow (Au^3+^) to purple (Au^0^).

Leaf extract from *P. daemia* was used as a reducer to produce Pd-AuNPs biogenically. Aqueous gold ions (Au^3+^) were reduced to gold nanoparticles (Au^0^ precipitate) by *P. daemia*. When biogenic AuNPs were successfully formed, the solution’s colour changed from yellow to deep purple (Figure 1b (insert)). The hydroxyl molecules, carbonyl compounds, and acid groups found in different phytochemicals are thought to be primarily responsible for the reduction mechanism. Even though plant-derived AuNP formation is well known, Pd-AuNPs made from *P. daemia* have special qualities that set them apart from other plant-sourced AuNPs, such as enhanced apoptosis induction via intrinsic mitochondrial reactions and specific cytotoxicity towards nasopharyngeal carcinoma cells [27], 28], 33].

### 3.2 UV–visible spectrum of biogenic Pd-AuNPs

UV–visible spectroscopy was used to measure the surface plasmon resonance with localization (LSPR), which additionally verified the production of AuNPs. Once activated by incoming light at a particular wavelength, the collective oscillations of free electrons bound to the surface of plasmonic nanoparticles are known as LSPR, a unique optical phenomenon associated with AuNPs [17]. Plasmonic nanomaterials’ unique characteristic makes it easier to use them in antibody-based assays, molecular image processing, and biological sensing [44]. In the visible spectrum, AuNPs’ LSPR manifests as a distinct absorbance band between 500 and 600 nm [45]. The obtained AuNPs showed absorption at *λ*_abs_ = 550 nm, as seen in Figure 1a, confirming that the AuNPs were successfully synthesized. The biogenically synthesised AuNPs’ size and shape determine the LSPR spectrum’s peak of absorbance and frequency. According to Figure 1a, the absorbance peak of our biogenically produced Pd-AuNPs at *λ*_abs_ = 550 nm suggests the formation of small-diameter spherical nanomaterials.

### 3.3 FT-IR analysis

**Figure 2:**
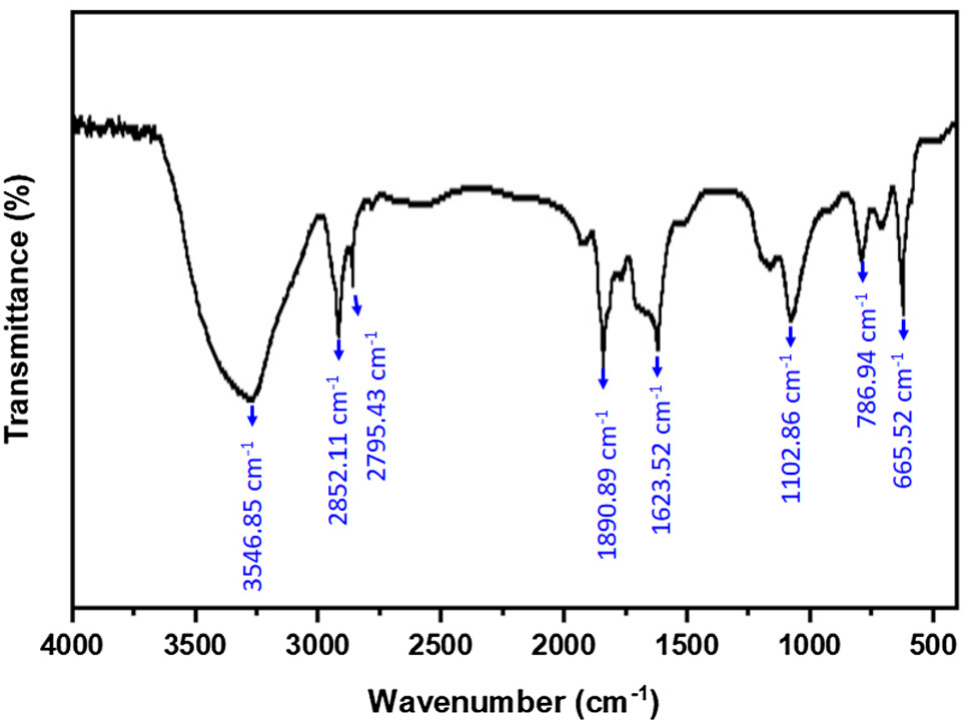
Fourier transform infrared (FTIR) spectra of biogenically synthesized Pd-AuNPs.

To assess the chemical constitution of the exterior of biogenic Pd-AuNPs and the local molecular atmosphere surrounding the capping molecules of the nanoparticles, FT-IR spectroscopy is employed. The existence of the phyto-materials that produce Pd-AgNPs was demonstrated by the FT-IR spectra in Figure 2. FT-IR study demonstrated that the phytochemicals, which make up the leaf extract, are both effective stabilizers and reducing agents. At 3,546.85 cm^−1^, the band represents O–H stretching vibrations. The amide region corresponds to absorption at 1,623.52 cm^−1^. These findings clarify the function of the *P. daemia* extract’s biological components in stabilizing AuNPs and lowering Au^3+^ [46]. The Pd-AuNPs FTIR spectra reveal distinct bands at 3,546.85 cm^−1^ and 2,852.11 cm^−1^ before and following the diminution of Au ions (Figure 2), which represent CH_2_ stretching vibrations and unattached O–H bonds, correspondingly. The polyphenols, proteins, tannins, and flavonoids that are present in *P. daemia* leaf extracts and serve as reducing agents for the creation of gold nanoparticles are all strongly indicated by such bands [47]. The ability of *P. daemia* leaf extracts to decrease and stabilize Pd-AuNPs was thus verified by the FT-IR spectroscopic analysis. The characterisation results unequivocally show that the current synthesis approach would be beneficial for synthesis and might resolve the long-standing issue of AuNPs stability for numerous purposes in treating cancer.

### 3.4 XRD pattern of the synthesized AuNPs

**Figure 3:**
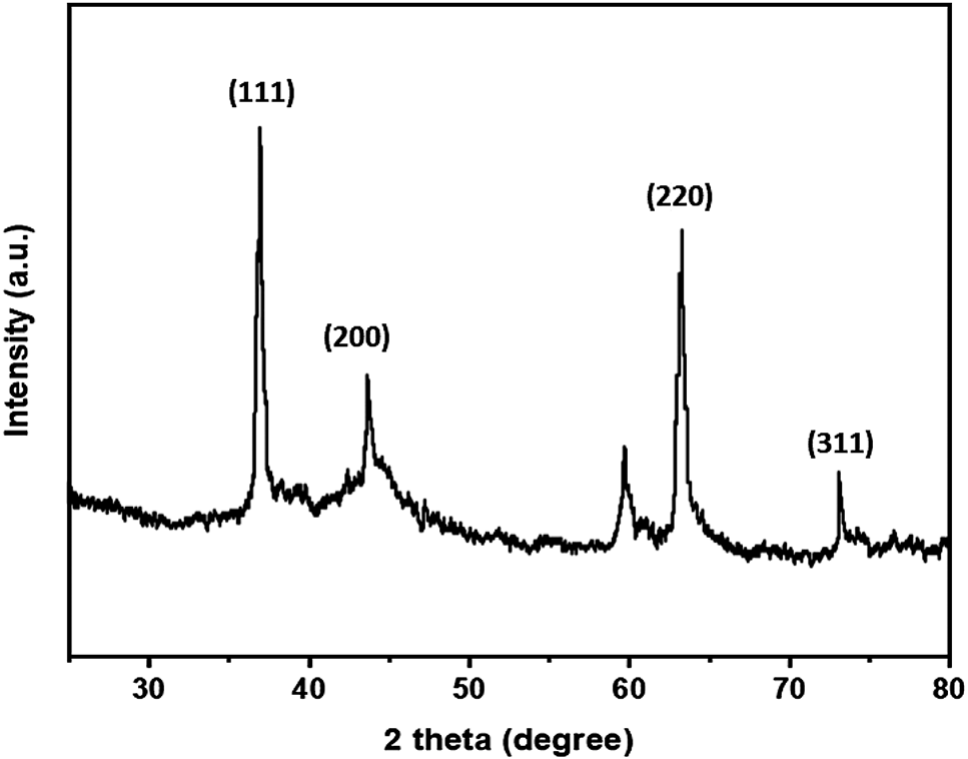
XRD pattern of Pd-AuNPs synthesized from *P. daemia*.

The crystalline structure of AuNPs was determined using the X-ray diffraction (XRD) method. At a 2̟θ angle, the scattered light pattern displayed eight distinct peaks, which were 38.20, 44.45, 64.62, and 77.51 (Figure 3). Most of the points line up with the Bragg reflections of the face-centered cubic (FCC) architecture (111), (200), (220), and (311), which is a common pattern for AuNPs and in line with other research on produced by biosynthesis AuNPs utilizing various extracts [48]. The diffraction peaks that were seen are the same as those that the ICD (00-004-0784) reports for standard gold metal [49]. The exceptional purity of AuNPs is further demonstrated by the lack of additional peaks. Therefore, there is compelling evidence from the pattern revealed by XRD that the AuNPs that were formed were made of pure crystallized gold NPs.

### 3.5 Morphology and EDX analysis of biogenically synthesized Pd-AuNPs

**Figure 4:**
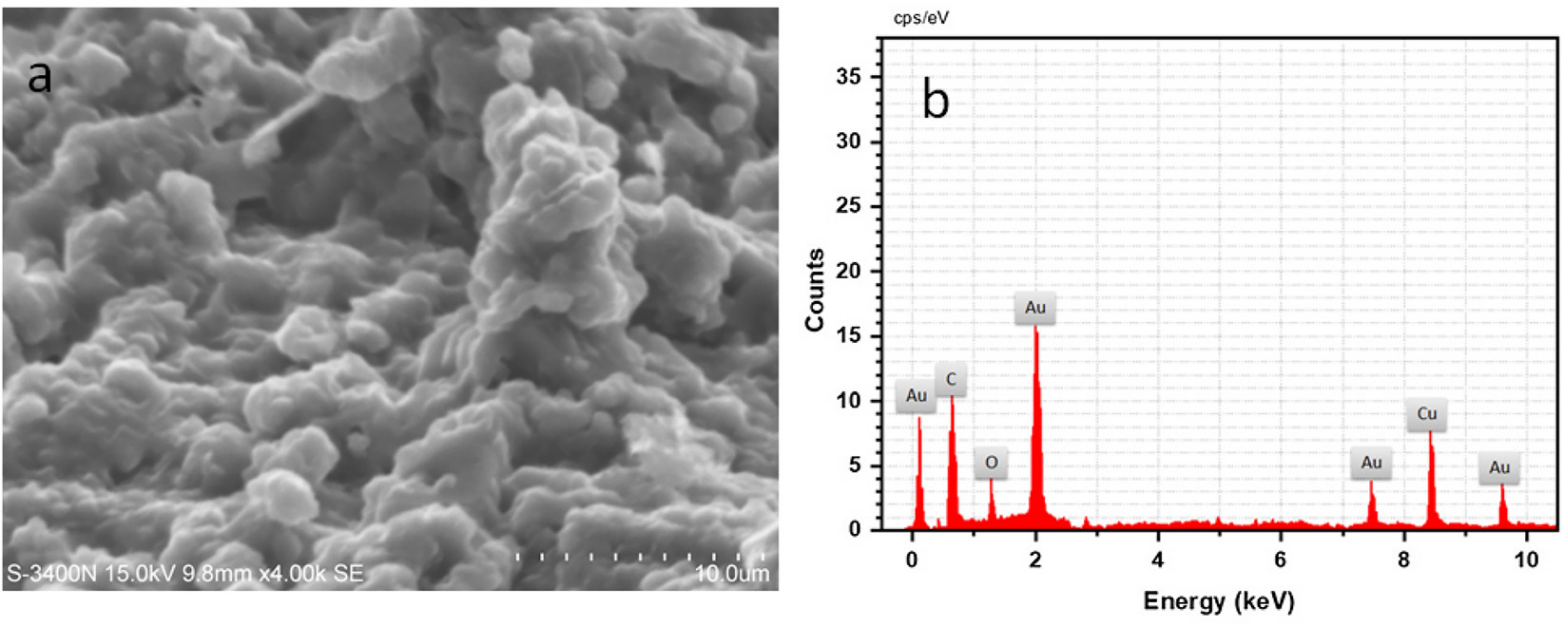
Analysing the physical attributes of particles involved as morphological investigation. (a) SEM images of biogenically fabricated Pd-AuNPs. (b) EDAX spectra of specific components in Pd-AuNPs are investigated.

FESEM was used to investigate the morphology of biogenically synthesized Pd-AuNPs (Figure 4a). As can be observed from the FESEM images (Figure 4a), biogenic Pd-AuNPs exhibited a nanosized morphology. The synthesized biogenic Pd-AuNPs showed an average diameter of approximately 110 nm. The chemical constituents of the extract often result in nanoparticles with varying sizes and shapes when using the biosynthetic approach [50]. The EDX analysis of the biogenically produced Pd–AuNP nanocomposite showed the presence of Pd, Au, and O elements (Figure 4b). A feature peak corresponding to gold absorption was observed at around 2.2 keV, which is characteristic of AuNPs [51], 52].

### 3.6 Diameter distribution and zeta potential analysis

**Figure 5:**
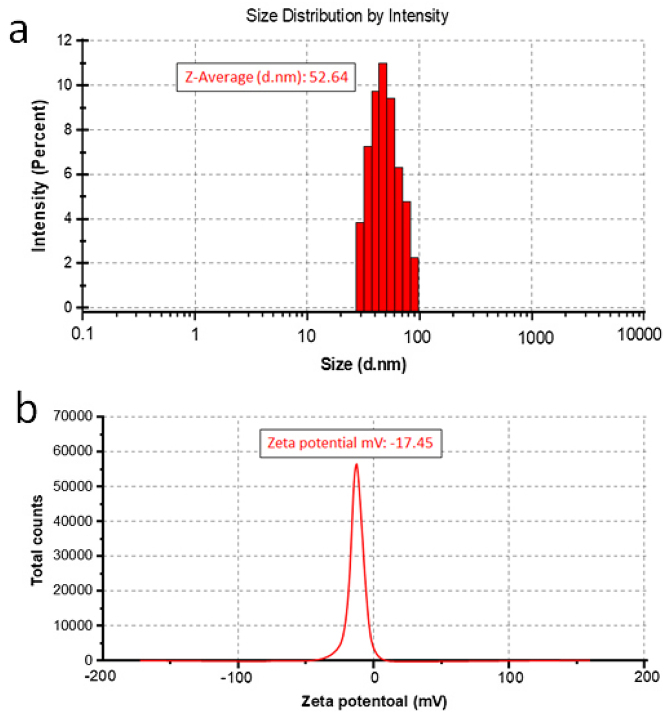
Dynamic light scattering (DLS) of *P. daemia-synthesised* Pd-AuNPs. (a) Size distribution and (b) zeta potential results of biogenically synthesized Pd-AuNPs.

The size distribution, agglomeration condition, and diminutive size of Pd-AuNPs were confirmed by dynamic light scattering (DLS). As seen in Figure 5a, our biogenically produced AuNPs were in fact approximately 53 nm in size according to DLS, whereas SEM imaging revealed aggregates with a diameter of about 110 nm. This apparent disparity can be explained by the differences between the two techniques. In contrast to SEM, which measures dried samples where particles may aggregate during the collection process, DLS evaluates the hydrodynamic diameter of nanoparticles in suspension, frequently producing larger values due to solvation layers while particles are uniformly distributed. Therefore, the greater apparent size from DLS is attributed to the fact that this technique measures the hydrodynamic diameter of agglomerated particles rather than the actual core nanoparticle size [53]. The AuNPs were found to be uniformly distributed, as indicated by a low polydispersity index (PDI) of 0.153.

To verify particle stability, zeta potential measurement was employed (Figure 5b). Generally, particles having a zeta potential above +30 mV or below −30 mV are regarded as stable in zeta potential studies [54]. Since the obtained value of −17.45 mV suggests moderate colloidal stability, it is possible that a certain quantity of aggregation could develop over time, even though the Pd-AuNPs remain well-dispersed. The observed dispersion stability of the colloidal solution may be attributed to the incorporation of phytochemicals from *P. daemia*, which probably aid in steric stabilization. Thus, despite the moderate zeta potential value, the phytochemical capping agents probably contribute to maintaining short-term colloidal stability (Figure 5b).

### 3.7 Cell viability assay

One of the key indicators for toxicological research is the cell viability assessment, which provides insight into how cells react to toxic compounds and reports on metabolic activity, cell death, and survivability, as well as how cells react to harmful substances. The effectiveness of natural anticancer plant extracts or chemicals may be assessed using a variety of techniques. The MTT test is one of the most widely used *in vitro* assays [55]. The most widely used test for determining the cytotoxicity of metal nanoparticles, plant extracts, versus both cancer and normal cell lines is the MTT experiment.

**Figure 6:**
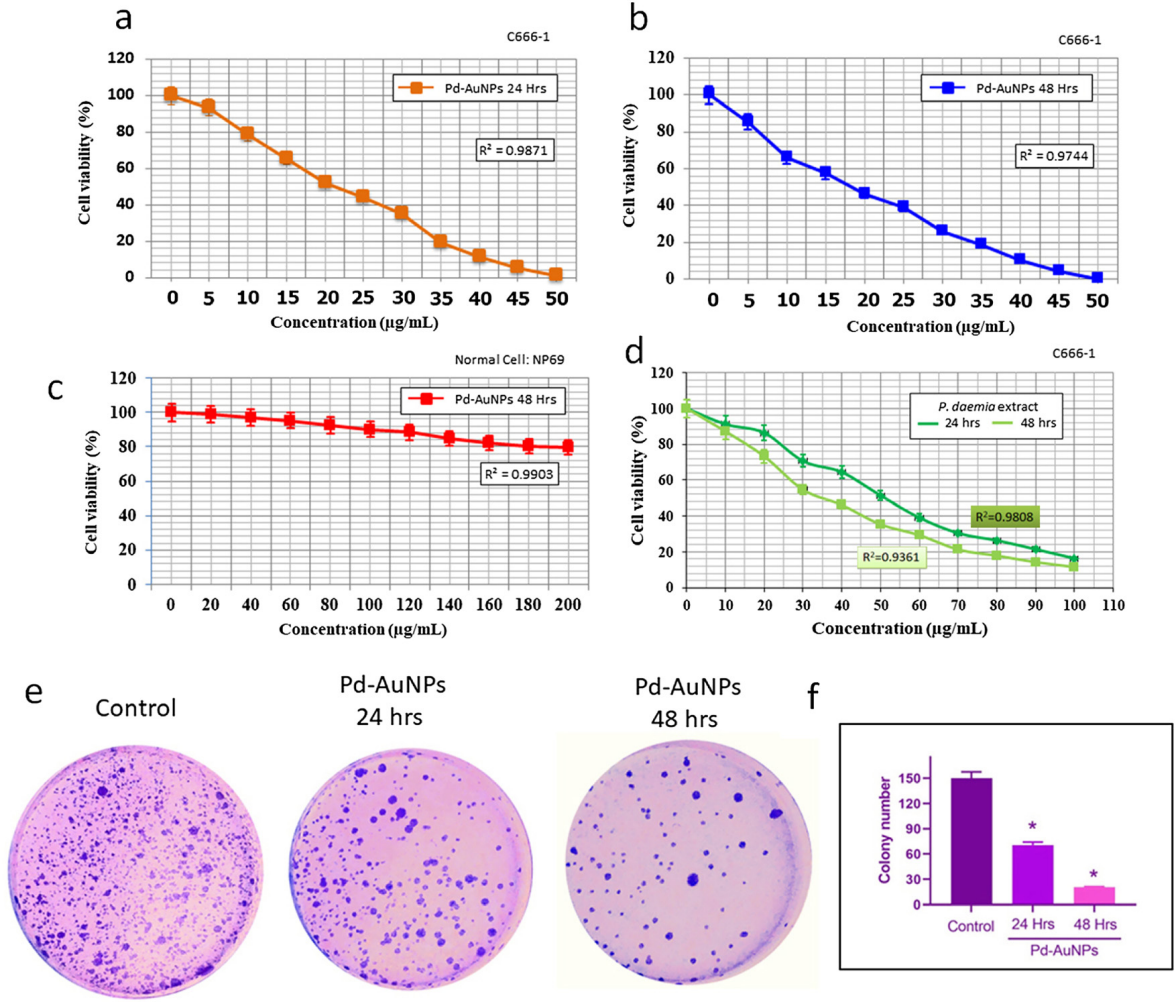
Cytotoxic and antiproliferative effects of Pd-AuNPs. (a) Dose- and time-dependent reduction in viability of C666-1 cells after 24 and 48 h of treatment. (b) Relative viability of NP69 normal epithelial cells after 48 h exposure (0–50 μg/mL). (c) NP69 cells maintained >85 % viability even at 200 μg/mL, confirming selective toxicity. (d) Cytotoxic effect of *P. daemia* leaf extract on C666-1 cells after 24 and 48 h of treatment. (e) Crystal violet staining showing decreased clonogenicity in C666-1 cells treated with IC_50_ concentrations of Pd-AuNPs for 24 and 48 h. (f) Quantification of colony counts. Data represent mean ± SD (*n* = 3); *p < 0.05 versus control (ANOVA).

The cytotoxicity of Pd-AuNPs in C666-1 and NP69 cells was investigated using the MTT test (Figure 6). Figure 6a and b illustrates how a higher Pd–AuNP concentration enhanced cytotoxicity after 48 h of incubation compared to 24 h. Furthermore, it was shown that the biogenically produced Pd-AuNPs had IC_50_ levels of 26.8 μg/mL at 24 h and 16.44 μg/mL at 48 h. Additionally, we have seen that Pd-AuNPs administration significantly reduced cell viability in C666-1 cells while increasing it in NP69 cells, suggesting that Pd-AuNPs are selectively toxic to tumour cells (Figure 6c). The IC_50_ values of biogenically generated Pd-AuNPs were used in additional studies, at 24 and 48 h accordingly. In contrast to other plant-produced AuNPs documented in studies, these findings demonstrate the novelty of Pd-AuNPs from *P. daemia* by highlighting their distinct bioactivity, exhibiting both dose-responsive cytotoxicity and particular action towards tumour cells [31], [32], [33]. One important component in determining the physiological function of NPs is the capping substance. By altering the capping molecules’ surface characteristics, such as charge, hydrophobicity, and functionality, their biological activity may be adjusted. Despite their effective endocytic absorption into human cells, AuNPs have been deemed innocuous by some researchers; nevertheless, different findings have been made about their cytotoxicity depending on their size, shape, and surface chemistry [56]. To improve stability and biocompatibility, we have included a novel *P. daemia* leaf extract, considered harmless and biocompatible [22], in the production of AuNPs.

To confirm the source of cytotoxicity, the crude *P. daemia* extract was independently tested for its influence on C666-1 cell viability within the same conditions of experimentation. The extract showed limited cytotoxicity (IC_50_: 50.9 μg/mL at 24 h and 35.4 μg/mL at 48 h), indicating that the significant, concentration- and time-dependent cytotoxicity observed is primarily due to the synthesised Pd-AuNPs rather than the plant-based chemicals independently (Figure 6d). According to these findings, all additional mechanism-based, apoptotic, and time-based investigations in the current investigation were conducted solely with Pd-AuNPs at their corresponding IC_50_ dosages. The low cytotoxic response of *P. daemia* substance alone when contrasted to Pd-AuNPs supports the hypothesis that the anti-tumour activity detected here is primarily owing to nanoparticle-dependent responses, with phytochemicals acting primarily as reducing and stabilising agents through the phases of biosynthesis.

### 3.8 Cell proliferation assay

Furthermore, the crystal violet assessment, which is displayed in Figure 6e and f, was used to analyse the treatment of biogenically produced Pd-AuNPs at the corresponding IC_50_ values linked to cell proliferation. Crystal violet staining is more noticeable in control cells, suggesting that cell proliferation is high. A moderate reduction in colony size and number was seen at 24 h (26.8 μg/mL), suggesting partial suppression of proliferative ability. On the other hand, cells treated for 48 h (16.44 μg/mL) showed a markedly reduced rate of nasopharyngeal cancer cell proliferation, indicating a persistent loss of clonogenic potential and a time-dependent increase in cytotoxic effectiveness. Remarkably, our results demonstrated that Pd-AuNPs may significantly reduce cancer cell growth *in vitro* and induce apoptosis in cancerous cells.

This prolonged exposure probably permits increased intracellular stress buildup, DNA damage, or persistent disruption of important cellular survival processes [57]. This suppression of clonogenic capacity has been linked in the past to interruption of cell cycle development and apoptosis mediated by nanoparticles [43]. Further evidence that Pd-AuNPs cause cytotoxicity not just through acute damage but also through persistent impairment of cellular repair and proliferating capacity comes from the higher suppression shown at 48 h. According to Nirmala et al. [58], these results are consistent with previous studies on the anti-clonogenic properties of other noble-metal nanoparticles, such as palladium- and gold-based nanocomposites, which have demonstrated promise in inhibiting tumour regrowth *in vitro*. As a result, the Pd-AuNPs reported in this work could be a viable nanotherapeutic approach for the long-term suppression of cancer cell growth.

### 3.9 Measurement of intracellular ROS level in biogenically synthesized Pd-AuNPs

**Figure 7:**
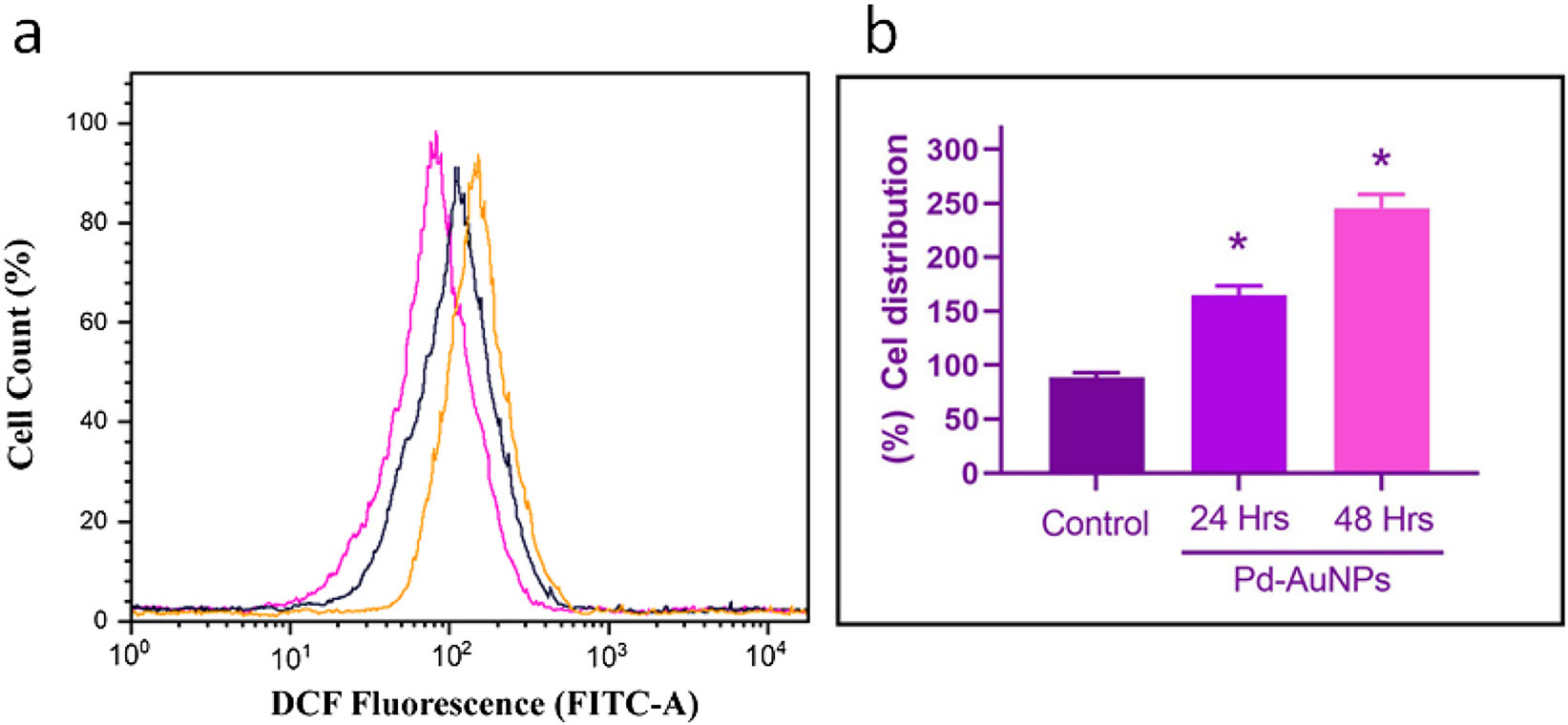
Intracellular ROS generation induced by Pd-AuNPs in C666-1 cells. (a) Flow cytometry analysis using DCFDA fluorescence. (b) Quantification of ROS levels. Data are presented as mean ± SEM (*n* = 3).

Once anticancer medicines are administered, one of the main biochemical changes in cancer cells is the generation of intracellular ROS, which is often increased and thought to be a factor that promotes cancer [59]. Therefore, employing spectrofluorimetry and flow cytometry, C666-1 cells were exposed to IC_50_ doses (26.8 and 16.44 μg/mL) of Pd-AuNPs for 24 and 48 h to measure the formation of ROS levels using DCFH-DA. Figure 7 shows the dose and time-dependent analysis of ROS generation after 30 min of Pd–AuNP treatment of C666-1 cells. When compared to untreated control cells, a significant change in DCF fluorescence intensity was observed after 24 h, suggesting increased ROS generation. At 48 h, a larger population of cells showed increased green fluorescence, indicating more ROS buildup; this rise was even more apparent. The fluorescent intensity values of C666-1 control cells were 100 %, but when cells were administered different levels of Pd-AuNPs, their levels changed. This indicates that C666-1 cells produced more ROS, which most likely caused apoptotic cell death via the mitochondrial-mediated process. Pd-AuNPs’ impact is demonstrated by the time-dependent rise in ROS in HeLa cells [59]. Our research yielded comparable outcomes.

### 3.10 DAPI staining

**Figure 8:**
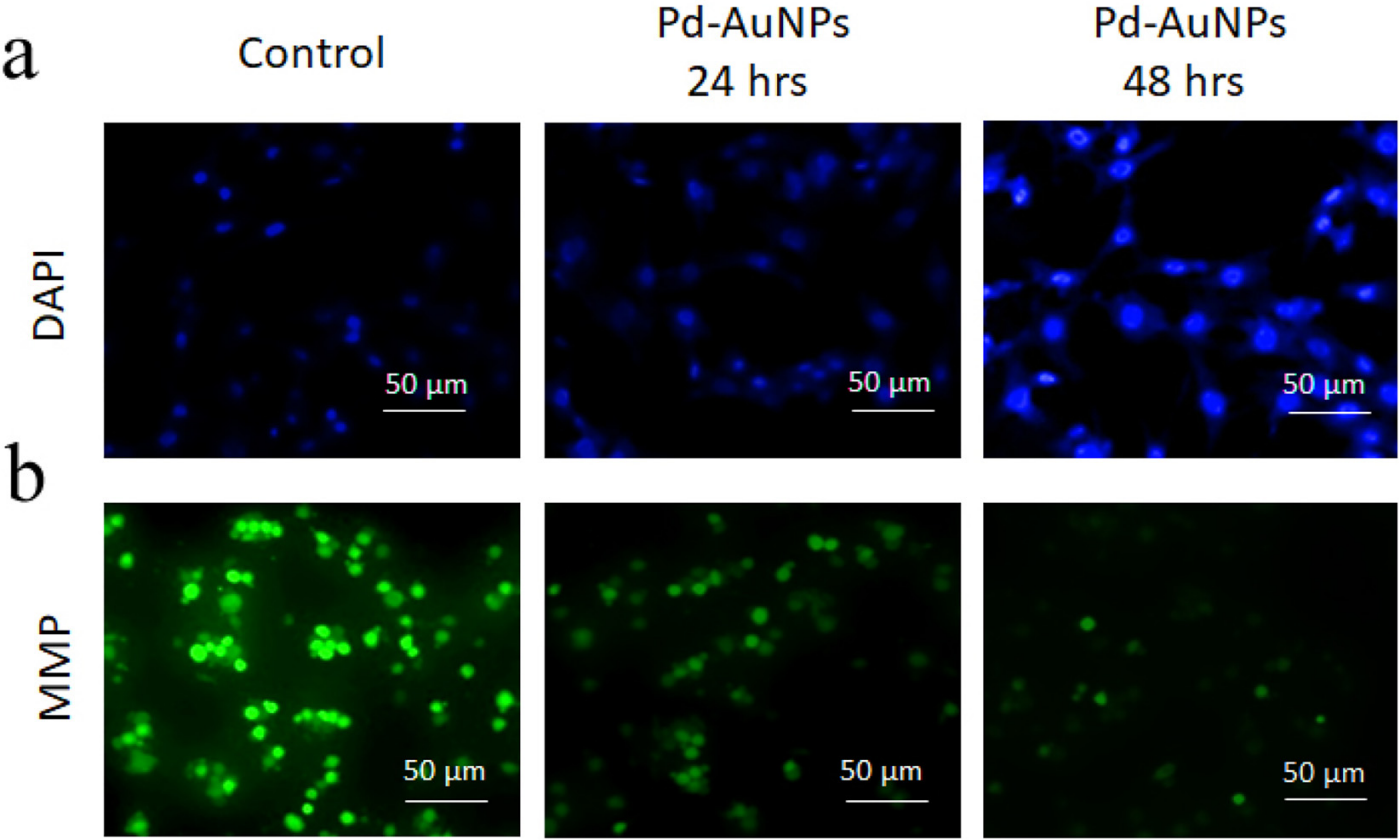
Pd-AuNPs effects on C666-1 cells. (a) DAPI-stained fluorescence images showing nuclear morphology after 24 and 48 h treatment with Pd-AuNPs at IC_50_. (b) Mitochondrial membrane potential (MMP) assessment using Rh-123 staining indicates decreased MMP in treated cells compared to control. Scale bar: 50 μm.

To investigate alterations in the nuclear architecture linked to apoptosis, cells treated with Pg-AuNPs at their respective IC_50_ concentrations (26.8 and 16.44 μg/mL) were incubated for 24 and 48 h before being labelled with DAPI. Cells showed signs of early apoptosis, including minor nuclear disintegration and chromosomal condensation, after being exposed for 24 h. In contrast, after 48 h, the cells treated with Pd-AuNPs displayed more apoptotic characteristics, including nuclear reduction, dispersed DNA, and condensed chromatin in the nucleus, which indicated cell death in an intensity- and time-dependent manner, while the control (untreated) cells displayed normal nuclei and unaltered cell membranes (Figure 8a). When using DAPI staining, the membrane-permeable dye specifically stains the nuclear debris of apoptotic cells, causing them to glow while the live cells remain unaltered. According to Uma Suganya et al. [60], exposure to biogenic AuNPs has been demonstrated to cause DNA damage and death in cancer cells, which is consistent with our findings.

### 3.11 Potential of the mitochondrial membrane (MMP)

A fluorescent microscope was used to find the MMP changes. In general, individuals are aware that apoptosis results in a drop in MMP. Rhodamine-123 staining was used to assess the biogenically produced Pd-AuNPs treatment, which was concerning since it relied on a change in MMP, an early sign of death. An early apoptotic signal was produced when C666-1 cells were exposed to Pd-AuNPs for 24 and 48 h at an IC_50_ concentrations, which affected the MMP (Figure 8b). An essential component of the intrinsic apoptotic process, the degradation of membrane potential of the mitochondria is a defining feature that initiates cell death. When biogenic Pd-AuNPs were administered to C666-1 cells, Rh123 staining revealed an interval- and dose-related decrease in Δ*Ψ*_m_. The findings presented here are consistent with other studies indicating that using oxidative stress-mediated mechanisms, biogenic CS-Pd/ZnO NCs, and gold nanoparticles damage the stability of mitochondria and induce apoptosis [43], 61]. According to the results, Pd-AuNPs target the mitochondrial metabolism to trigger intrinsic apoptosis signalling.

### 3.12 Dual staining assay

**Figure 9:**
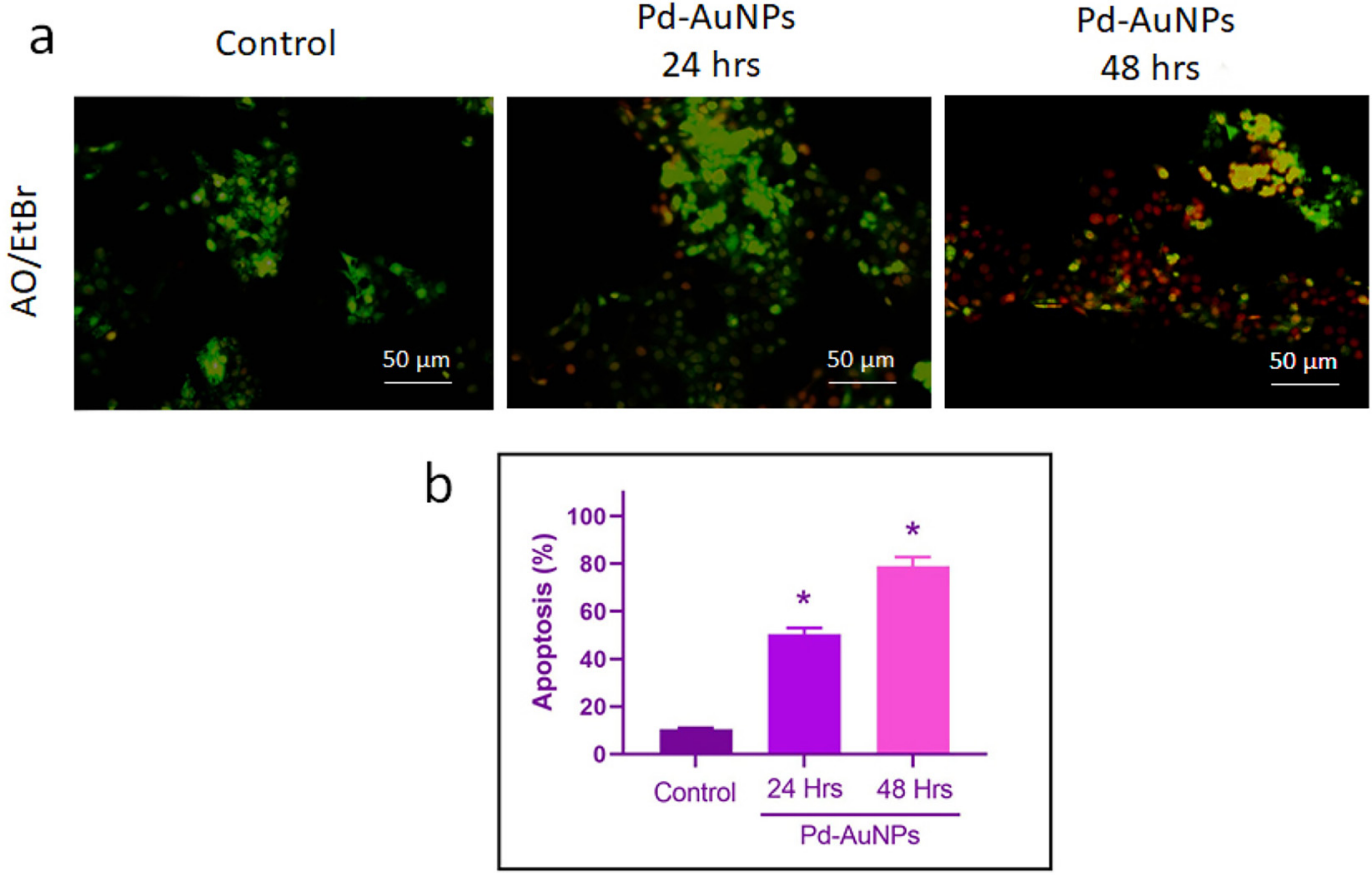
Pd-AuNPs induce apoptosis in C666-1 cells. (a) AO/EB fluorescence images showing increased early and late apoptotic cells after 24 and 48 h treatment with Pd-AuNPs at IC_50_, indicated by yellow/orange fluorescence, membrane blebbing, DNA fragmentation, and chromatin condensation. Scale bar: 50 μm. (b) Quantification of apoptotic cells (%), expressed as mean ± SD (*n* = 3). *p < 0.05 versus control.

Apoptosis is a constitutionally regulated process that causes cell death in a stepwise manner. AO/EtBr staining for fluorescence microscopy was used to compare the detection and measurement of necrotic and apoptotic cells. This process depends on the different uptake of the Ao/EtBr fluorescent DNA-binding dye. After 24 and 48 h of being administered with IC_50_ values of biogenically produced Pd-AuNPs (26.8 and 16.44 μg/mL), we measured cell mortality by analyzing AO/EtBr staining (Figure 9). Apoptotic cell counts increased in an interval and dose-related manner, according to fluorescence microscopy analysis. At a dosage of 16.44 μg/mL, Pd-AuNPs caused AO/EtBr-positive cells in C666-1 cells to undergo apoptosis after 48 h. This was an up-regulation of the apoptotic percentage, and the majority of the cells were positive for AO/EtBr staining, displaying morphological alterations in contrast to control cells. The cells treated with Pd-AuNPs showed features of apoptosis, such as cell reduction, nuclear condensation, disintegration, and the appearance of apoptotic bodies in AO/EtBr staining, while the control cells displayed uniform vibrant green nuclei and the cytoplasm for AO stains and barely positive cells in EtBr stain. The number of apoptotic cells increased significantly over time, according to quantitative results (Figure 9b). Following treatment with Pd-AuNPs, the proportion of apoptotic cells increased from 15 % in the control group to 57 % at 24 h and then to 80 % at 48 h (*p < 0.05). Our current investigation concludes that Pd-AuNPs may induce cell death in C666-1 cells through mitochondrial pathways controlled by ROS, which triggers apoptotic events. This suggests that Pd-AuNPs are effective preventative and therapeutic agents [62]. The results highlight the advantages of Pd-AuNPs as a possibly advantageous therapy that inhibits proliferation and induces cell death.

### 3.13 Pd-AuNPs induce apoptosis in C666-1 cells

**Figure 10:**
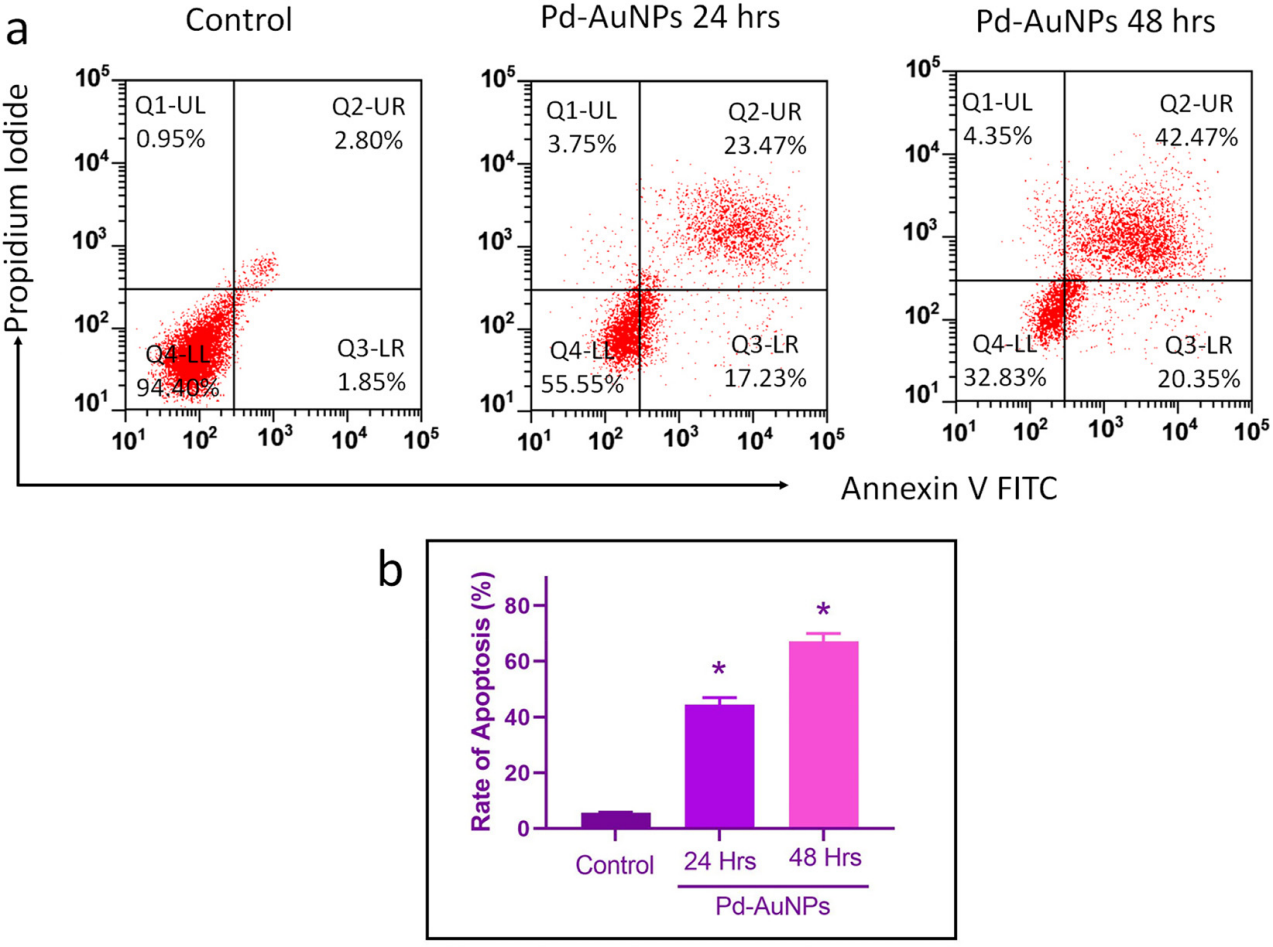
Flow cytometric analysis of apoptosis using annexin V/PI staining. (a and b) Flow cytometric analysis of apoptosis in C666-1 cells. Cells were stained with PI/Annexin V to assess early and late apoptotic populations following Pd–AuNP treatment, statistically significant distinction (*p < 0.05 vs. control) expressed as mean ± SD (*n* = 3).

In order to strengthen the evaluation of cell apoptosis, PI/Annexin V flow cytometry was used. Biogenically synthesized Pd-AuNPs were added to C666-1 cells at concentrations of 26.8 and 16.44 μg/mL, respectively. As shown by the flow cytometric probe in Figure 10a and b, the ratio of apoptotic cells increased from 5.60 % in control cells to 44.45 % after 24 h and 67.17 % after 48 h of Pd-AuNPs treatment. The statistical significance of these changes (*p < 0.05) indicates that Pd-AuNPs induced apoptosis in C666-1 cells effectively.

### 3.14 Pd-AuNPs’ impact on cell cycle arrest

**Figure 11:**
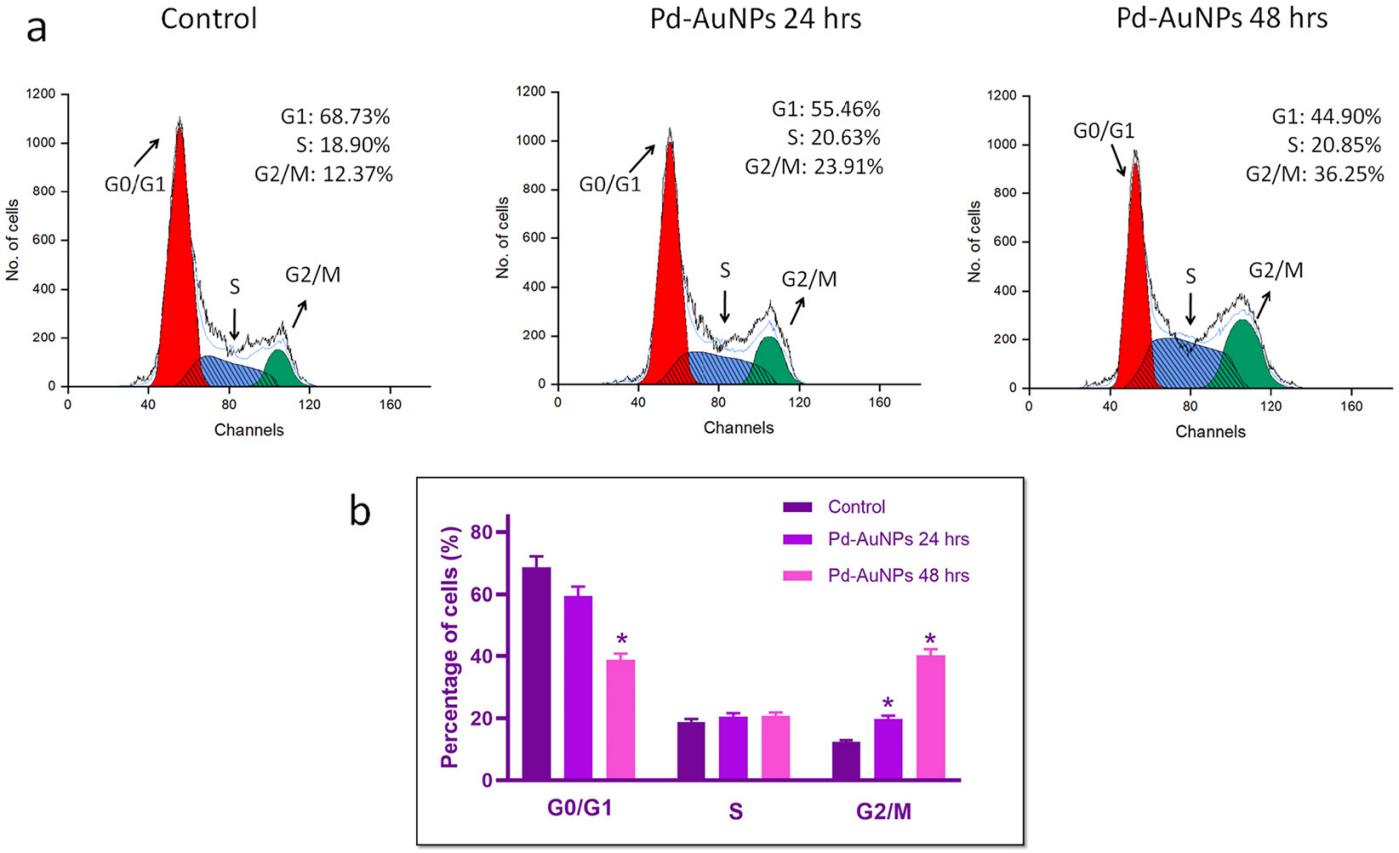
Effect of Pd-AuNPs on cell cycle progression of C666-1 cells. (a and b) Effect of Pd-AuNPs on cell cycle distribution of C666-1 cells. Statistically significant distinction (*p < 0.05 vs. control) expressed as mean ± SD (*n* = 3).

Flow cytometry examination of Propidium Iodide (PI)-stained cells was carried out following a 24 and 48-h Pd–AuNP therapy to examine the effect of Pd-AuNPs on the cell cycle. In comparison to control cells, the Pd-AuNPs-treated C666-1 cells showed a significant accumulation of cells during the G2/M phase (Figure 11a and b). This finding suggests that Pd-AuNPs prevent C666-1 cells from proliferating by causing cell cycle arrest at the G2/M phase.

### 3.15 Modulation of apoptosis-associated genes in C666-1 cells using biogenic Pd-AuNPs

**Figure 12:**
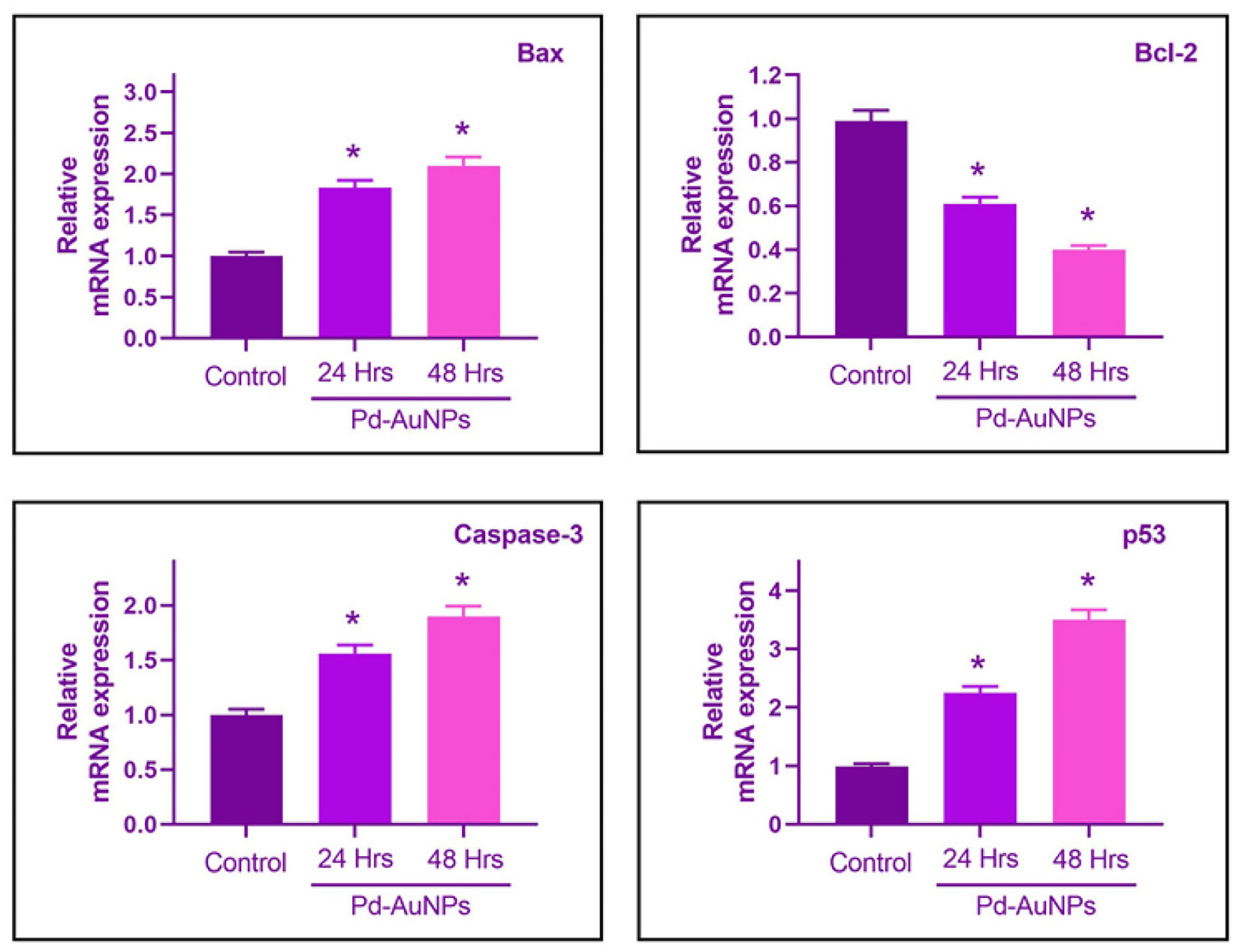
Pd-AuNPs modulate apoptosis-related genes in C666-1 cells. RT-PCR analysis showing increased expression of (a) Bax, (c) caspase-3, (d) p53, and (b) decreased expression of Bcl-2 following Pd–AuNP treatment.

Alterations in mRNA levels of important apoptotic genes were measured using qPCR analysis in order to comprehend the molecular mechanism of antitumor action [41]. Pro-apoptotic Bax, Caspase-3, and p53 genes were dramatically upregulated by 2.5, 2.1, and 2.9-fold, respectively, over the untreated control after 24 and 48 h of treatment with an IC_50_ dose of Pd-AuNPs (Figure 12). At the same time, the anti-apoptotic Bcl-2 gene, which prevents cell death, was substantially decreased by over 5–6 times, indicating that the intrinsic mitochondrial pathway induces apoptosis. The idea that Pd-AuNPs cause mitochondrial breakdown and apoptosis based on caspase is supported by the elevated Bax/Bcl-2 proportion and caspase-3 activity. The findings presented here are consistent with other research showing comparable apoptotic signals caused by nanoparticles in different instances of cancer [63], 64]. Pd-AuNPs from *P. daemia* fill a research gap in plant-derived nanoparticles by combining specific cytotoxicity, ROS-mediated mitochondrial impairment, and modulation of apoptosis-induced genes to create a unique and potentially beneficial nanotherapeutic substance for nasopharyngeal carcinoma [34], 35].

**Figure 13:**
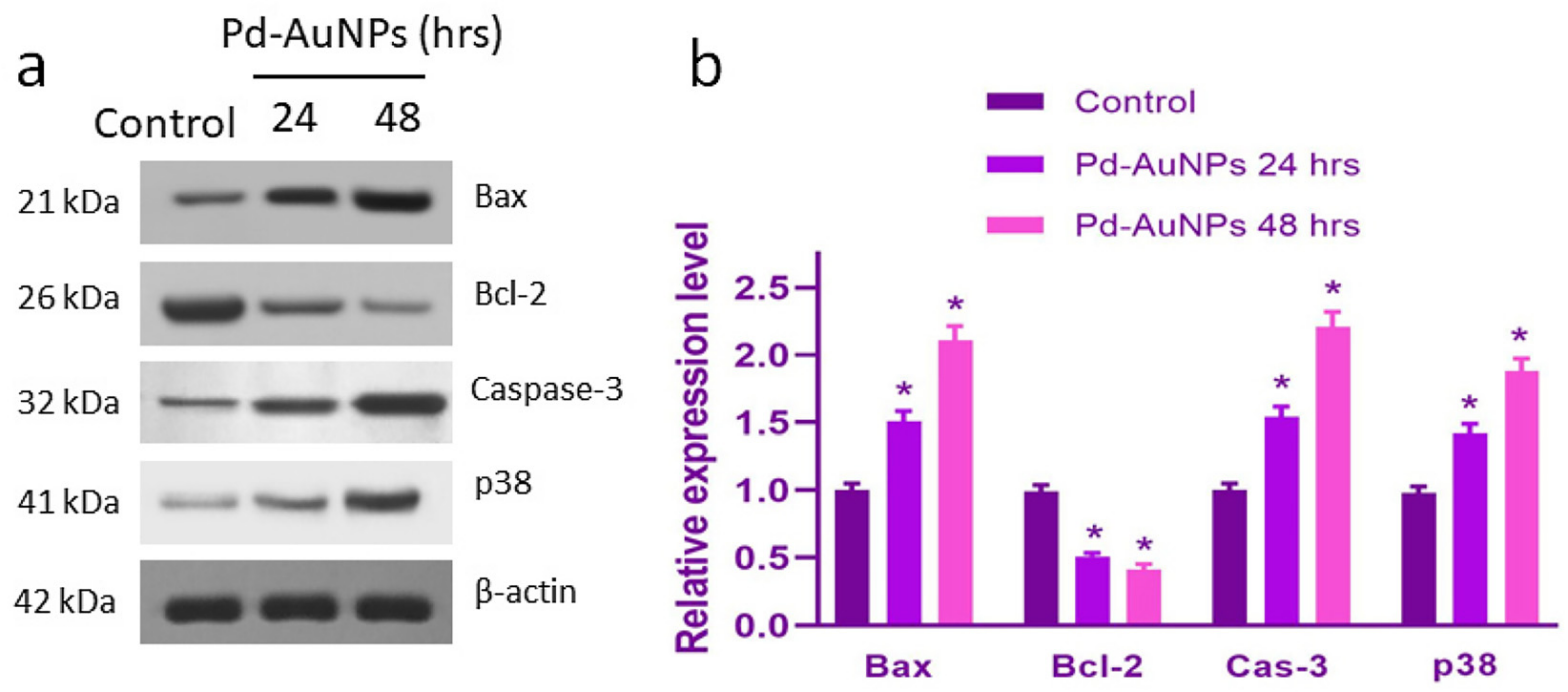
Western blot analysis of apoptosis-associated proteins in C666-1 cells treated with Pd-AuNPs. Data are expressed as mean ± SD (*n* = 3). Statistical analysis was performed using one-way ANOVA with Tukey’s multiple comparison test; *p < 0.05 versus control.

Western blot evaluation of the most important indicators of apoptosis, including Bax, Bcl-2, Caspase-3, and p53, was carried out to confirm these transcriptional assessments at the protein level (Figure 13a and b). In comparison to the control group, Pd–AuNP-treated C666-1 cells showed a clear increase in the abundance of Bax, Caspase-3, and p53 proteins, as well as a substantial reduction of Bcl-2 protein. The results of this study are consistent with the RT-PCR results, confirming that Pd-AuNPs cause apoptosis by activating the intrinsic mitochondrial pathway.

Overall, our results suggest that Pd-AuNPs derived from the extracts of *P. daemia* leaves induce apoptosis in C666-1 cells by triggering mitochondria-mediated apoptosis. Experimentally, Pd-AuNPs enhanced the formation of ROS, which led to mitochondrial membrane depolarization; Bax, caspase-3, and p53 were upregulated, whereas Bcl-2 was downregulated. This results in caspase-mediated apoptotic cell death. In contrast to previous publications on AuNPs targeting widespread tumours, this is the first study to show the potential beneficial effects of Pd-AuNPs against nasopharyngeal carcinoma. This work is novel and has translational relevance because, to our knowledge, this is the first publication to link Pd-AuNPs with apoptosis induction in C666-1 cells via the intrinsic mitochondrial pathway.

Although this study provides valuable data regarding the therapeutic properties of biogenic Pd-AuNPs in C666-1 cells, there are several drawbacks. Because the current study was limited to *in vitro* analysis, further investigation, including *in vivo* experiments and a comprehensive evaluation of molecular pathways such as cell cycle regulation and various signalling cascades, was not carried out. These aspects will be addressed in future studies to provide a more complete understanding of the therapeutic potential of Pd-AuNPs.

## 4 Conclusions

We developed dependable, biodegradable gold nanoparticles with leaf extract from *P. daemia*. By capping the outermost portion, the phytochemicals in the substance stabilized the nanoparticles and effectively converted gold ions to AuNPs. Human nasopharyngeal carcinoma C666-1 cells showed encouraging *in vitro* anticancer potential from the biogenically synthesized Pd-AuNPs. After 24 and 48 h, their IC_50_ values decreased from 26.8 to 16.44 μg/mL, indicating a dose and time-dependent substantial inhibition of cancer cell growth. The loss of cell viability and distinct intracellular damage were seen under a microscope. Additionally, biogenically synthesized Pd-AuNPs altered the quantities of mRNA implicated in apoptosis according to quantitative real-time PCR measurement. In summary, our findings suggest that biogenically synthesized Pd-AuNPs may induce apoptosis through modulation of p53, Bax/Bcl-2, and caspase mechanisms *in vitro*. This provides preliminary evidence supporting the role of the mitochondrial intrinsic pathway in apoptosis induction in nasopharyngeal carcinoma cells. Accordingly, the current work proposes *P. daemia*-mediated biogenic synthesis as an easy, environmentally responsible method of creating AuNPs with potential *in vitro* anticancer applications. However, these findings are limited to *in vitro* studies; further investigations, including pharmacokinetic profiling, safety evaluation, and *in vivo* validation, are essential before any therapeutic significance can be established.
